# Increasing the chance of dying at home: roles, tasks and approaches of general practitioners enabling palliative care: a systematic review of qualitative literature

**DOI:** 10.1186/s12875-023-02038-0

**Published:** 2023-03-23

**Authors:** Shangavi Balasundram, Anne Holm, Kirstine Skov Benthien, Frans Boch Waldorff, Susanne Reventlow, Gritt Overbeck

**Affiliations:** 1 The Research Unit for General Practice and Section for General Practice, University of Copenhagen, Copenhagen, Denmark; 2 Palliative Care Unit, Copenhagen University Hospital, Hvidovre, Copenhagen, Denmark

**Keywords:** Palliative care, General practice, Systematic review, Primary care

## Abstract

**Background:**

Many elderly people wish to die at home but end up dying at the hospital. If the patient wishes to die at home, palliative care provided by General Practitioners (GPs) may increase the chance of dying at home, however, there is a lack of knowledge on how GPs should provide palliative care. We aimed to identify roles, tasks and approaches of GPs enabling palliative care, by exploring the experiences of GPs, other healthcare professionals, patients, and relatives through a systematic review of the qualitative literature.

**Methods:**

We searched PubMed, EMBASE, PsycINFO, Web of Science, and CINAHL in March 2022. Thematic analysis was used for synthesizing the results.

**Results:**

Four thousand five hundred sixty three unique records were retrieved, and 12 studies were included for review. Of these, ten were interview or focus group studies and two were survey studies with additional open-ended questions. Only qualitative findings from the studies were used in synthesizing the results. Thematic analysis produced four main themes describing the roles, tasks and approaches of GPs enabling palliative care to increase the chance for patients to die at home. GPs can support patients in the final phases of life by applying a holistic, patient-centred**,** and proactive approach to palliative care and by having sufficient education and training. Furthermore, the palliative care consultation should include symptom management, handling psychosocial and spiritual needs, maintaining a fragile balance, and proper communication with the patient. Lastly, GPs must address several palliative care elements surrounding the consultation including initiating the palliative care, being available, being the team coordinator/collaborator, providing continuous care and having sufficient knowledge about the patient.

**Conclusions:**

The roles, tasks and approaches of the GPs enabling palliative care include being aware of elements in the palliative care consultation and elements surrounding the consultation and by having sufficient education and training and a broad, proactive, and patient-centred approach.

**Supplementary Information:**

The online version contains supplementary material available at 10.1186/s12875-023-02038-0.

## Background

Dying is the inevitable outcome of living but talking about death can still be taboo for both patients and their General Practitioner (GP). When elderly people are asked about their preferred place of death, the majority wish to die at home [1, 2]. Despite the wish of dying at home, most of the elderly people die in the hospital [3]. Rapid and unexpected deterioration of the patient’s health may lead to a patient dying in the hospital even if they wish to die at home, but the three following factors may prevent unwanted hospitalization; 1) that the patient has not been identified as needing palliative care, 2) that the physician caring for the patient is not comfortable providing palliative care and 3) because the preferred place of death has not been discussed with the patient and their relatives before it is too late.

To help physicians identify patients with palliative care needs, several tools with different complexity have been developed, including SPICT [4], the Surprise Question [5] and RADPAC [6]. Palliative care is a practice that attempts to prevent and relieve suffering for patients with life-threatening diseases by improving the quality of life [7]. Different concepts within palliative care have emerged, including end-of-life care and advance care planning [8–11]. In this paper, we use the term palliative care as an umbrella term covering both end-of-life care and advance care planning. After the identification of patients with palliative care needs, the GP is in a central position to discuss death and provide palliative care in case the patient wishes to die at home. However, several studies have demonstrated physicians’ self-perceived barriers in providing palliative care, including a lack of skills and confidence in several significant factors, including addressing cultural and spiritual needs [12, 13]. To manage terminal patients to increase the quality of dying, different models in general practice have been developed, that establish safe organizational pathways for the dying and their relatives. In the Netherlands, models have been developed, where groups of GPs and district nurses have regular interprofessional meetings with support from a palliative care consultant to discuss specific and present patient issues [14], and in several countries, both GPs and palliative care specialists provide palliative care [15–17]. Generally, there has been an increased focus on palliative care for patients with cancer diagnoses [18]. However, for patients with organ failure illnesses as seen in old age, palliative care has not been studied as widely. Furthermore, previous studies have suggested and tested methods to promote GPs’ competencies in palliative care [14, 19] and a lot of studies have been made on GPs’ challenges and barriers in providing palliative care [20–23], but there is a lack of equivalent focus on factors enabling GPs in providing palliative care. The aim of this exploratory systematic review of qualitative literature was to identify roles, tasks and approaches of GPs enabling palliative care, by exploring the experiences of GPs, other healthcare professionals, patients, and their relatives.

## Methods

A systematic review of the qualitative literature was performed. We used Enhancing Transparency in Reporting the synthesis of qualitative research (ENTREQ) standards [24].

### Search strategy and sources

We developed a comprehensive search strategy in collaboration with an information specialist. Following a PICO format, we formed a list of search terms within these categories 1) General practitioner; 2) Palliative care; 3) Patient Preference/psychology and synonyms hereof (see the Supplementary Data for the search string). We searched the following databases on the 18^th^ to the 25^th^ of March 2022: PubMed, EMBASE, PsycINFO, Web of Science, and CINAHL for studies published between March 2012 and March 2022. Studies recommended by experts were included and we manually searched the references list of the included studies to make sure no relevant articles were missing from the search.

### Study selection

One author (SB) conducted a preliminary screening, identifying, and removing duplicates as well as discarding articles obviously not meeting the aim of our research question (e.g., paediatrics, oncology, euthanasia), based on titles and abstracts. Two authors (SB and GO) then screened the remaining articles separately according to the inclusion criteria (see below). If no abstract was available, and the title was of interest, the full text was screened. After having reached an agreement upon which articles to read in full text, the two authors, in collaboration, agreed on which to include in the review.

#### Inclusion of studies

Our inclusion criteria were as follows:

- 1. Studies published in English between March 2012 and March 2022, based on original qualitative data (mixed methods studies were included if the qualitative element contributed data about GPs’ experiences with palliative care).
- 2. Studies exploring the views and experiences of GPs, other healthcare professionals, patients, and their relatives on roles, tasks and approaches of GPs enabling palliative care, without interest in one specific diagnosis, training or education, and after the identification of palliative care needs.
- 3. Studies had to have health care systems in which the GP is the first point of contact and thereby act as a gatekeeper to specialist care, including specialized palliative care.

### Quality assessment

The first author (SB) employed the consolidated criteria for reporting qualitative research checklist (COREQ) to assess the quality of transparency in the included studies [25]. Questions that arose from this process were discussed and resolved with the last author (GO). We neither excluded, nor gave special priority to any studies during the analysis based on the COREQ quality assessment. Survey reports with an addition of qualitative data were not assessed because COREQ would not be applicable in these studies, and assessment tools for survey studies do not assess the qualitative data of the survey reports.

### Data extraction and synthesis of results

The aim of this review was exploratory, which made it relevant to apply an inductive analysis method. No ‘a priori’ theory or framework was used, and a thematic analysis of the findings of the included studies was performed.

Thematic analysis is a recognized and widely used method for identifying, analyzing, and reporting patterns (themes) within qualitative data [26]. Data were extracted from the results of the studies.

First, the author (SB) performed a pilot coding of themes of one of the studies. Subsequent studies were coded into pre-existing themes, and new themes were created when deemed necessary.

Thereafter the sub-themes were categorized into actual themes relevant to the research question. The coding was done with the free online software program Notion [27].

## Results

### Study selection

**Fig. 1:**
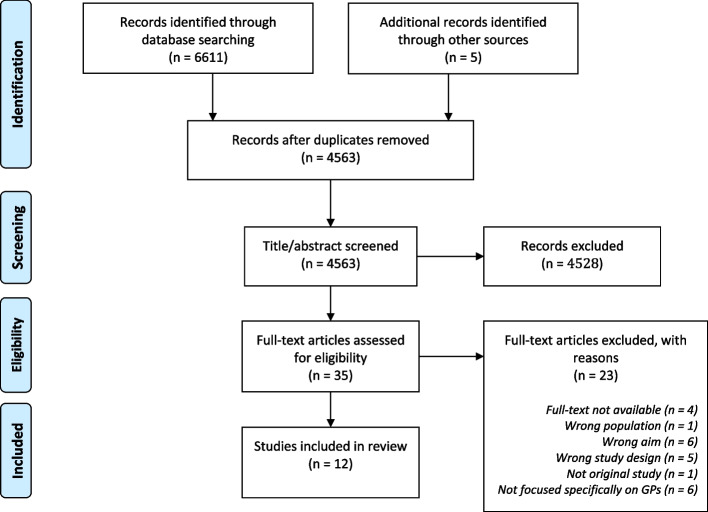
Flow chart of study selection

Study selection is depicted in Fig. 1. The search identified 6611 references, of which 2049 were identified as duplicates. Screening of titles and abstracts resulted in the selection of 35 articles for a full-text assessment of eligibility. For four articles, the full texts were not available, and the first author was contacted, though we received no replies. Out of the 35 articles, 23 were excluded, mainly because of (1) not focused specifically on GPs (*n* = 6), (2) wrong aim (*n* = 6) or (3) wrong study design (*n* = 5); 12 studies were included in our final review. The references within those articles were also checked for potentially eligible articles, though no new studies were identified.

### Study characteristics

Table 1 shows the characteristics of the 12 studies we included. The studies were from 7 different countries: Germany (*n* = 3), the UK (*n* = 2), Canada (*n* = 2), Australia (*n* = 2), Belgium (*n* = 1), Portugal (*n* = 1), the United States (*n* = 1). Six of the 12 studies were interview studies, three studies used focus groups, and one was a combination of both focus groups and interviews. The last two studies were survey studies with the addition of open-ended questions. The majority of the studies used thematic analysis and content analysis. One study included critical incident technique before the analysis, one used grounded theory, one used framework analysis and the two survey studies also included closed-ended questions in questionnaires. In those cases data were included descriptively even though the methodology was quantitative.

**Table 1:** Study characteristics

First author with reference	Year of publication	Country (ISO 3)	Study objectives	Study description	No. of participants	Description of participants	Methodology and analysis
Beernaert et al. [28]	2015	BEL	To explore the views of family physicians, nurses, and patients about the tasks of the family physician in palliative care for people with a life-limiting illness from diagnosis onwards	Focus groups and semi-structured interviews	50	18 interviews with patients 6 focus groups, 4 with FPs (*n* = 20) and 2 with community nurses (*n* = 12)	Thematic content analysis
Cardoso et al. [29]	2021	PRT	To understand the perspectives of specialist and trainee family physicians about their role in palliative care	Focus groups	19	Two focus groups; one with 10 family physician trainees and one with 9 family physicians	Thematic analysis
Geiger et al. [30]	2016	DEU	To explore the tasks and challenges regarding the care for frail older patients in the last phase of life from the GPs’ point of view, and the latter’s perception of their own role and responsibilities	Semi-structured interviews	14	GPs	Grounded Theory
Herrmann et al. [31]	2019	AUS	To explore Australian GPs’ perceptions of barriers and enablers to the provision of palliative care and provides new insights into how to implement best practice care at the end of life	Semi-structured interviews	25	GPs	Content analysis
Herrmann et al. [32]	2019	AUS	To explore, in a sample of Australian GPs, their perceptions of best practice palliative care and their ideal role in its delivery	Semi-structured interviews	25	GPs	Content analysis
McCallan et al. [33]	2021	CAN	To (1) explore the barriers family physicians encounter in providing palliative and end-of-life care in our metropolitan context and (2) identify potential strategies to overcome these challenges	Interviews	10	Family physicians	Thematic analysis
Mitchell et al. [34]	2016	GBR	To provide insight into the experience of GPs providing End-of-Life care in the community, particularly the facilitators and barriers to good-quality care	Questionnaire survey	516	GPs	Descriptive statistics and thematic analysis
Rewegan et al. [35]	2019	CAN	To explore how a palliative approach to care is operationalized in primary care, through the description of clinical practices used by primary care clinicians to identify and care for patients with progressive life-limiting illness (PLLI)	Semi-structured interviews	11	6 physicians, 3 nurse practitioners, 1 registered nurse, and 1 registered practical nurse	Content analysis
Sharp et al. [36]	2018	GBR	To investigate the attitudes of GPs to advance care planning discussions with frail and older individuals	Focus groups	21	GPs	Framework analysis
Silveira et al. [37]	2012	USA	To explore the factors influencing primary care providers' ability to care for their dying patients in Michigan	Focus groups	50	16 focus groups; twenty-eight primary care providers and twenty-two clinical support staff	Thematic analysis
Stiel et al. [38]	2020	DEU	To explore positive and negative experiences in PC in Germany from the perspectives of patients, relatives, and health care professionals in a primary care setting	Interviews	16	Patients, relatives, GPs, medical assistants, and nurses	Critical Incident Technique and thematic analysis
van Baal et al. [39]	2020	DEU	To evaluate the quality of End-of-Life Care from a GP’s perspective using the German version of the General Practice End-of-Life Care Index	Survey and qualitative questions	52	GPs	Descriptive statistics and content analysis

*AUS* Australia, *BEL* Belgium, *CAN* Canada, *DEU* Germany, *GBR* Great Britain *PRT* Portugal *USA* the United States

### Quality assessment

The explicitness and comprehensiveness of the ten interview studies were assessed using the COREQ checklist [25] (see Supplementary data). Substantial heterogeneity in reporting items was found between studies, with a range of 11–29 (out of 32) reported items with both a median and mean of 18.

The domain in which transparency (openness about the analytical process) was found to be most lacking was the research team and reflexivity; only two studies stated the experience and training of the research team members [28, 37], and only one described interviewer characteristics [28]. One study did not describe the sampling method [33] and two studies lacked a description of the sample [36, 37]. The ten interview studies were transparent in reporting by presenting quotations and data. Furthermore, the findings were consistent and there was a clarity of major themes [28–33, 35–38].

### Synthesis of results

**Fig. 2:**
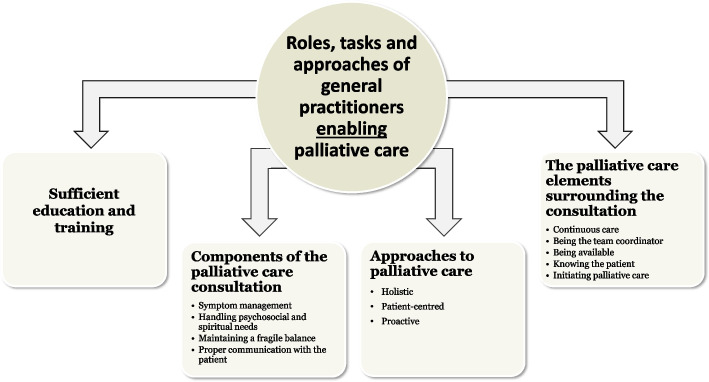
Schematic model of the roles, tasks and approaches of general practitioners enabling palliative care

The included studies described experiences from physicians, other healthcare professionals, patients, and their relatives on palliative care provided by the GPs. Across the included studies we identified 4 main themes describing the roles, tasks, and approaches, that enable GPs in providing palliative care: one about the approaches to palliative care, a second about education and training, a third about the palliative care consultation, and a fourth about elements of palliative care surrounding the consultation. The four themes contained several subthemes visualized in Fig. 2. The themes were synthesized based on all the qualitative findings from the included 12 studies.

#### Approaches to palliative care

Several of the included studies stressed the importance of a holistic approach to palliative care [28, 30, 32, 38]. In one study the participating GPs, nurses and patients described the main role of the GP as to relieve the patient from physical symptoms and provide psychological and existential care [28]. In another study one GP reported her own experience with a holistic approach to care for a patient:

“We took over a patient from a GP. And he was a patient suffering from end-stage COPD, who additionally had a pulmonary embolism and in consequence a failure of a large part of his lung. The whole hallway, the entrance area, was full of oxygen bottles and the man was lying in a small room. The man had his oxygen running at 14 litres. So we came there and slowly got to know each other. We explained everything during our talk, for a while, and we touched upon his matters. The oxygen was lowered to 4 litres. This was very impressive, that’s when we noticed this had a psychosocial component.” [38]

In combination with the holistic approach, several studies pointed out the relevance of being proactive [28, 32, 35, 38]. This should be done because it gives the patient a sense of control and important care decisions can be made before it is too late [32]. A way of doing so is to plan appointments for patients ahead of time, as one GP does:

“We often intentionally book patients’ appointments for them, I mean, you know a lot of appointments are made when the patient perceives there’s a problem… but we often switch to intentionally booking appointments when we feel there’s discussions that need to take place around their care. [35]

Furthermore, the importance was stressed to a patient-centred approach [31, 32], which preserves the patient’s autonomy [30]. As one GP put it:

‘You know whatever way they want to go I just let them know that they’re in the driver’s seat and I’m just there to facilitate it for them.’ [32]

#### Sufficient education and training

The GPs in the studies had different opinions about education on palliative care. Some GPs judged they had enough experience and/or training [31], whilst others expressed a need for further education [29, 31, 39], preferably as “hands-on learning” and mentoring [31]. In one study GPs and nurses stressed that it is the responsibility of the GP to stay informed about palliative care and that this could be done through training courses [28]. In another study, GPs pointed out the need for training by categorizing three subjects: clinical training, communication and understanding of the network [29].

#### Tasks and contents of the palliative care consultation

One of the tasks of the GPs in providing palliative care is to care for the patient’s symptoms [28, 29, 31, 39]. As one GP told:

“…Undividable part of his role as [a] doctor” (the ability to manage less complex symptoms)[29]

Another task is to care for the patient’s psychosocial and spiritual needs [28, 30, 32], and for the fragile elderly persons, being attentive to changes in symptoms and psychosocial needs will help maintain a “fragile balance”, which could prevent hospitalisation [30]. As one GP described an elderly couple:

“They [the frail couple] are at high risk of falling, highly vulnerable to everything. […] They are supported by all the props our system has. More is not possible. It’s all in a state of fragile balance!”[30]

Furthermore, a role enabling palliative care is being able to communicate properly with the patient [28, 30, 32, 39], this includes active listening, realistic communication about life expectancy and asking patients about their wishes [32, 38]. Illustrated below with an example from a GP:

“Sometimes we don’t even know if the patient is informed or not [about the diagnosis], that’s the worst thing. We received phone calls saying, “Yes, yes, but my dad is not supposed to know about it [meaning end-of-life stage]”, or nope, the wife doesn’t want her husband to know, then I say, “Then we actually can’t care for him”. When we get involved, we do insist that patients are made aware. And we deal with that [palliative care] in rare cases because of the [patients’] huge distress. No one else can provide it [palliative care without patients being informed about diagnosis/prognosis], but we still do it... reluctantly. And if we were to stay at the residents’ bedside, we would speak the truth. Anything else would make no sense. Everybody should question themselves,“ [Do I want] to be fooled by my wife, husband, children [...]?”. The end is near, maybe someone wants to arrange something, perhaps you would like to put all your thoughts in order. I think you might be deprived of your life and the very last part of it, that’s a shame.” [38]

#### The palliative care elements surrounding the consultation

##### Initiating palliative care

After the identification of palliative care needs, the next step is to initiate palliative care. GPs stressed that early in the patient care trajectory they should be the facilitator for discussions about end-of-life care and that patients should be allowed to plan care by giving their prognosis earlier than the very final stage. The knowledge of their prognosis will also lead to better practice care in the later stages of illness [32]. As for advance care planning, an option is to ‘plant seeds’ [36], which allows the patient to think about it and discuss it when they are ready:

“I might not say all at once, right, we need to discuss a plan for you, but you just mention it when you might see them every 2 months or something.”[36]

##### Being available

In several of the included studies, GPs stressed the importance of being available [28, 31, 32, 39]. By having a flexible schedule GPs can make time for unplanned visits and be available in case of emerging needs [28, 37]. Other ways of being available are by using telephone and online communication [31], or by enabling patients to book appointments on short notice [37]. This would prevent the patients from going to urgent care:

“[Urgent care is] used because [patients] might not be able to get an appointment with a provider… [A problem] becomes even more urgent and they just go to urgent care.”[37]

As time and illnesses progress, patients get more limited. As a result, and as a part of being available the GP’s task consists of offering home visits [30–32, 35]. One GP talked about her experience with home visits, stressing that mostly, but not all the time, home visits are for patients near the very end of life:

“For some people I book regular home visits – if they’re at that point when patients are finding it difficult to come and see me, and often it is more near the end of life, but not necessarily. I had one lady who I was doing home visits for, for the last two years of life. Just because she couldn’t come and see me, so that was the best way to do it.” [35]

Furthermore, one GP hired a social worker, to manage the coordination of care, lessening the pressure on the GP and being fully available for patients [37].

##### Being the team coordinator/collaborator

All the included studies described the significance of GPs being a collaborator or a coordinator [28–39]. The involvement of others includes the family, the other practice personnel, specialists, other professionals, and if relevant district nurses.

Several studies pointed out the function of a GP as a coordinator [28, 31, 32, 35, 37, 39]. Where GPs can delegate caring needs [28], as one GP put it:

“(…) the fact that we are being told, does not mean we have to solve it .... We can offer paths, options or give people advice. [28]

Another option is to have palliative care nurses support day-to-day care [31]. And when the end is near it is the responsibility of the GP to involve other professionals e.g., specialist palliative home care [28, 39].

Because GPs often are the patients’ first point of contact, GPs see themselves as the ones leading the patient’s care [32]. This could be done by leading multidisciplinary teams [29] and being the one leading at the very end of life [28].

The function of GPs as a collaborator was repeatedly stressed [31, 32, 34, 35, 38, 39]. Facilitators of collaboration are communication between GPs and homecare nurses [33, 34], and information exchange between involved healthcare professionals both medical and non-medical [28, 30, 31]. An online communication platform between patients, multidisciplinary healthcare professionals and relatives/supportive others was suggested [31].

Another way of being the team coordinator proposed by multiple GPs is by involving the other practice personnel [30, 31, 35, 37]. This is because palliative care is not only medical but could also include other care practices, such as wound care [30]. Delegation of wound care would result in regular check-ins with other practice personnel which could potentially lead to a decrease in appointments with the GP self. As one GP explained it from her own experience:

“And we actually have very [well] qualified practice assistants who also partly take over home visits. […] And we also have three wound managers […] we are very happy, that it’s not just left to the nursing agencies, but that someone from the practice has a look at least once a week.” [30]

Furthermore in managing and supporting patients with palliative care needs, the involvement of the family is valuable for palliative care [28, 30, 35, 36, 39], including the GP’s assessment of how much of a caring capacity is available for the patient [28]. Having the family involved early on would enable more straightforward discussion leading to fewer difficulties in the future [36]. One GP described her need for involving the family:

“That’s why I naturally make regular visits to these very old [patients], to see, when the time comes, which [of the family members] I may now also involve. Whom can I rely on? Firstly, to understand what the patients themselves want, but also to understand what concerns those standing beside them.” [30]

In one study it was proposed to have a family member designated as a primary caregiver who could come to every appointment [35]. Another study stated that life-limiting illness affects both the patient and their family [28], thereby pointing out that GPs should involve and communicate with the family as well [28].

##### Continuous care

The relationship between the GP and the patient enables the GP in providing palliative care. One important factor in the relationship is continuity of care [30, 32, 35, 37]. As one GP put it:

“Actually, (I see that) also as my obligation as a GP, because they know me and so they will then also ask for me. […] So I can’t fade away when it comes to the end!” [30]

The continuity of care enables decision-making and planning for End-of-Life [37]. Furthermore, GPs in one study claimed that the best palliative care was long-term and ongoing [32].

##### Knowing the patient

Another task that enables palliative care is to know the patient [36, 37]. As an example, it would make it easier for GPs to initiate advance care planning [36]. It would also lead to deeper trust, which is also important in decision-making [37]. Additionally, one GP meant it is important to concretize the relationship for the patient, with the result of being capable of making better decisions:

“I tell them outright, whatever happens, good or bad, I’m going to be there for you… I am going to do my best to help you. I try to get emotionally involved, because you’re caring for people, and you’ll make better decisions because you’ll know where they’re coming from”[35]

## Discussion

To our knowledge, this is the first systematic review of qualitative studies on roles, tasks and approaches enabling GPs to provide palliative care.

These can be divided into four themes; approaches to palliative care; education and training; contents of the palliative care consultation; and elements surrounding the consultation. The roles, tasks and approaches of GPs enabling palliative care are a holistic, patient-centred and proactive approach, being sufficiently educated and trained, focusing on the following contents of the palliative care consultation; symptom management; handling psychosocial and spiritual needs; maintaining a fragile balance; proper communication with the patient, and addressing the following palliative care elements surrounding the consultation: initiate palliative care, be available, be the team coordinator/collaborator, provide continuous care and know the patient.

Various models to enhance palliative care provided by GPs have been evaluated [14, 19, 40–42]. One of them described the development of the Patz group consisting of GPs, district nurses and a palliation specialist as the facilitator of the collaboration [14]. Patz groups aim at enabling collaboration between healthcare professionals as was identified in this review as an important task for the GP in providing palliative care. Another model was a combination of an educational meeting and an electronic decision support that provided GPs with relevant knowledge to help the GP track the patient’s status in end-of-life and to address relevant topics [40]. This is in accordance with our findings as it supported having sufficient education and training and facilitating GPs in providing better palliative care. Furthermore, a pathway with 8 elements, including assessment of the patient’s needs; medication review; multidisciplinary meetings; and good coordination and communication, has also been tested [19], addressing several of the themes addressed in this review. Lastly, a narrative review described the roles of the GP in providing end-of-life care [43] and found in concordance with our findings, that the GPs need to address the patient’s symptoms, and social, emotional and spiritual needs and manage information and coordinate and collaborate.

### Strengths and limitations

A major strength of this review is the rigorous methodology that was used during the search. The systematic and thorough approach, developed with the aid of an information specialist, was used to develop the search string and was used in five major databases. To ensure contemporary relevance of the synthesis we limited the search to a 10 year period. All reference lists from the studies included in this review were examined to ensure no relevant articles were missing. Another strength is that after the first screening and removal of duplicates, two authors in collaboration selected the studies included in the review. Furthermore, we used the ENTREQ standards for reporting qualitative reviews, and a quality assessment was made of the qualitative data using COREQ. The inclusion of studies based on participants’ experiences is also a strength because it gives qualitative data that reflect real-life enablers of palliative care. However, some limitations apply: Only limited qualitative research in this area has been published. We excluded studies published in other languages than English and before 2012 and our research question is not the same as in the included studies. Furthermore, the use of quality standards does not necessarily guarantee the quality of intellectual work where interpretation is essential. More specifically, while COREQ does capture several relevant aspects of quality, in our opinion, it also has some limitations as an assessment tool due to its strong focus on the formal aspects of research papers and its relatively less focus on analytical content.

It was a strength that the twelve studies were conducted in seven different countries, strengthening the results of this review to be applicable in many countries. Secondly, the participants in the studies were a variation of GPs, nurses, patients, and their relatives leading to a differentiated view of GPs’ work. Thirdly, the studies were a mix of focus groups and independent interviews, covering more topics than one of the study methods could have provided.

Although, we also identified some limitations of the included studies: Firstly, the COREQ assessment of the quality of the studies showed a variation in scores between 11 and 29 out of the 32 criteria. Secondly, the included studies did not use participant observations. These could strengthen the validity of findings by supporting the participants’ statements from the interviews but might be costly and impractical.

### Implications

Findings from this review suggest roles, tasks and approaches of GPs that enable palliative care. Further research needs to be done to identify other enablers, that are not identified in this review. A focus on enablers instead of barriers will make the subject more likely to enter our consciousness and make it easier for GPs to practice in everyday work.

This study proposes a schematic model of the roles, tasks and approaches of GPs enabling palliative care (Fig. 2), which can be used to reduce the complexity of palliative care provision.

All of the included studies stressed the importance of collaboration between the involved medical professionals, including specialists of relevance. Thereby stressing the importance of developing a system that facilitates communication between medical professionals so collaboration can be effective, and thereby enhancing the palliative care for patients.

## Conclusion

To increase the chance for patients to die at home, GPs can support patients in the final phases of life by applying a holistic, patient-centred, and proactive approach to palliative care and by having sufficient education and training. Furthermore, the palliative care consultation should include symptom management, handling psychosocial and spiritual needs, maintaining a fragile balance, and having proper communication with the patient. Lastly, GPs must address several palliative care elements surrounding the consultation including initiating the palliative care, being available, being the team coordinator/collaborator, providing continuous care and having sufficient knowledge about the patient.

## Supplementary Information

**Additional file 1.** Search string.

**Additional file 2.** COREQ Checklist.

## Data Availability

All data and materials used during the present systematic review are available from the corresponding author.

## Abbreviations

GP: General practitioner
ENTREQ: Enhancing transparency in reporting the synthesis of qualitative research
COREQ: Consolidated criteria for reporting qualitative research
