# Supplementary material for: Increasing the chance of dying at home: roles, tasks and approaches of general practitioners enabling palliative care: a systematic review of qualitative literature
Source: BMC Prim Care. 2023 Mar 23;24:77. doi: 10.1186/s12875-023-02038-0 (PMC10035229; doi:10.1186/s12875-023-02038-0)
Supplement: Supplementary file 1 — Additional file 1. Search string. [file 12875_2023_2038_MOESM1_ESM.docx]

# Supplementary data

## Search string

Identification of search terms and thesauruses for the chosen concepts

|  | **Identified search terms** | **Thesauruses** |
| --- | --- | --- |
| **#1 Subject**  Search title/abstract | "Palliative Care" OR "Terminal Care" OR “Preferred place of death” | **Pubmed:**  "Palliative Care" + "Terminal Care"  **Embase:**  “Palliative therapy” + “Terminal care” + “Advance care planning”  **PsycInfo:**  “Palliative care” + “Terminal Care”  **CINAHL:**  “Palliative Care” + “Terminal Care”  **Web of Science:** N/A (no MESH-terms) |
| **#2 Setting**  Search title/abstract | "General Practic*" OR "generalist*" OR "Family Physician*" OR "Primary Care Physician*" OR "GP*" OR "PCP*" | **Pubmed:** "General Practice" + ("General Practitioners" + "Physicians, Family" + "Physicians, Primary Care"  **Embase:**  general practice/ + general practitioner/  **PsycInfo:**  “General Practice” + “General Practitioners” + “Physician, Family” + “Physicians, Primary Care”  **CINAHL:**  “Family Practice + “Physicians, Family”  **Web of Science:** N/A (no MESH-terms) |

Search string used in the individual databases

|  | **Date and time** | **Search** | **No. of hits** |
| --- | --- | --- | --- |
| **Pubmed** | 18/03-2022, 11:15 | ((((("Palliative Care"[Mesh]) OR ("Palliative Care")) OR ("Terminal Care"[Mesh])) OR ("Terminal Care")) OR ("Preferred place of death")) AND ((((((((((("General Practice"[Mesh]) OR ("General Practic*")) OR ("General Practitioners"[Mesh])) OR ("generalist*")) OR ("Physicians, Family"[Mesh])) OR ("Family Physician*")) OR ("Physicians, Primary Care"[Mesh])) OR ("Primary Care Physician*")) OR ("GP*")) OR ("PCP*")) AND (y_10[Filter]) | 1995 |
| **Embase** | 25/3-2022, 13:40 | (palliative therapy/ or terminal care/ or advance care planning/ or ("palliative care" or "terminal care" or "preferred place of death")) and (general practice/ or general practitioner/ or ("General practic*" or "Family physician" or "Generalist*")) and limit to yr="2012 -Current" | 2542 |
| **PsycInfo** | 25/3 - 2022, 13:55 | (MA (“Palliative care” OR “Terminal Care”) OR (Palliative care” OR “Terminal Care” OR “Preferred place of death”)) AND (MA (“General Practice” OR “General Practitioners” OR “Physician, Family” OR “Physicians, Primary Care”) OR (“General Practic*” OR “Family Physician*” OR “Primary care physician*” OR “Generalist*”)  Limit to last 10 years | 807 |
| **CINAHL** | 25/3-2022, 14:10 | ((MH “Palliative Care”) OR (MH “Terminal Care+”)) OR (Terminal Care” OR “Palliative Care” OR “Preferred place of death”) AND ((MH “Family Practice) OR (MH “Physicians, Family”) OR “General Practic*” OR “Generalist*” OR “Family Physician*” OR “Primary Care Physician*”)  Limit to last 10 years | 425 |
| **Web of Science** | 25/3-2022, 14:30 | (“Palliative care” OR “Terminal Care” OR “Preferred place of death”) AND (“General practic*” OR “Family physician*” OR “Primary care physician*” OR “Generalist*”  Limit to last 10 years | 848 |
