# Supplementary material for: Increasing the chance of dying at home: roles, tasks and approaches of general practitioners enabling palliative care: a systematic review of qualitative literature
Source: BMC Prim Care. 2023 Mar 23;24:77. doi: 10.1186/s12875-023-02038-0 (PMC10035229; doi:10.1186/s12875-023-02038-0)
Supplement: Supplementary file 2 — Additional file 2. COREQ Checklist. [file 12875_2023_2038_MOESM2_ESM.docx]

## COREQ Checklist

|  | Number of studies that reported the criterion | | References to the studies |
| --- | --- | --- | --- |
| **Domain 1: Research team and reflexivity** | | | |
| 1. Interviewer/facilitator | 6 | 60% | [29, 30, 34-37] |
| 2. Credentials | 8 | 80% | [29-31, 33-37] |
| 3. Occupation | 3 | 30% | [29, 30, 35] |
| 4. Gender | 1 | 10% | [29] |
| 5. Experience and training | 2 | 20% | [29, 30] |
| *Relationship with participants* | | | |
| 6. Relationship established | 2 | 20% | [29, 30] |
| 7. Participant knowledge of the interviewer | 2 | 20% | [29, 30] |
| 8. Interviewer characteristics | 1 | 10% | [29] |
| **Domain 2: study design** | | | |
| *Theoretical framework* | | | |
| 9. Methodological orientation and Theory | 10 | 100% | [29-38] |
| *Participant selection* | | | |
| 10. Sampling | 9 | 90% | [29, 30, 32-38] |
| 11. Method of approach | 8 | 80% | [29, 30, 32-37] |
| 12. Sample size | 10 | 100% | [29-38] |
| 13. Non-participation | 2 | 20% | [35, 36] |
| *Setting* | | | |
| 14. Setting of data collection | 9 | 90% | [29, 30, 32-38] |
| 15. Presence of non-participants | 3 | 30% | [29, 30, 33] |
| 16. Description of sample | 8 | 80% | [29, 31, 33-38] |
| *Data collection* | | | |
| 17. Interview guide | 7 | 70% | [29, 32-34, 36-38] |
| 18. Repeat interviews | 0 | 0% | None |
| 19. Audio/visual recording | 10 | 100% | [29-38] |
| 20. Field notes | 1 | 10% | [29] |
| 21. Duration | 8 | 80% | [29, 30, 32-37] |
| 22. Data saturation | 6 | 60% | [29, 31, 33, 35-37] |
| 23. Transcripts returned | 1 | 10% | [29] |
| **Domain 3: analysis and ﬁndings** | | | |
| *Data analysis* | | | |
| 24. Number of data coders | 8 | 80% | [29-31, 34-38] |
| 25. Description of the coding tree | 2 | 20% | [29, 34] |
| 26. Derivation of themes | 10 | 100% | [29-38] |
| 27. Software | 5 | 50% | [29, 30, 32, 34, 37] |
| 28. Participant checking | 2 | 20% | [29, 37] |
| *Reporting* | | | |
| 29. Quotations presented | 10 | 100% | [29-38] |
| 30. Data and ﬁndings consistent | 10 | 100% | [29-38] |
| 31. Clarity of major themes | 10 | 100% | [29-38] |
| 32. Clarity of minor themes | 2 | 20% | [33, 38] |
